# Allyl sulfide incorporated polymethyl methacrylate and tricalcium phosphate fibrous composites for osteoblast proliferation investigation

**DOI:** 10.3389/fbioe.2026.1792824

**Published:** 2026-06-01

**Authors:** Xunkai Hou, Mariappan Rajan, Anne Seles Mohan, Suresh Mickymaray, Tao Bai

**Affiliations:** 1 Department of Trauma and Hand and Foot Surgery, Shandong Provincial Third Hospital, Cheeloo College of Medicine, Shandong University, Jinan, China; 2 Biomaterials in Medicinal Chemistry Laboratory, Department of Natural Products Chemistry, School of Chemistry, Madurai Kamaraj University, Madurai, India; 3 Department of Biology, College of Science- Al Zulfi, Majmaah University, Al Majmaah, Saudi Arabia; 4 Department of Orthopedics, Yan’an People’s Hospital, Yan’an, China

**Keywords:** allyl sulfide, bioactive composite, osteoporosis, polymethylmethacrylate, β-tricalcium phosphate

## Abstract

Osteoporosis is a systemic bone disorder characterized by a disparity in bone restructure, leading to decreased bone strength and worsening microstructure. Among bioactive compounds incorporated into ceramics, recent attention has focused on the repair or regeneration of osteoarthritis-affected bone. β-tricalcium phosphate (β-TCP) remains a widely used material for bone regeneration due to its excellent biocompatibility and the ability of bone cells to restore it. In this study, a novel composite of β-TCP reinforced with Polymethylmethacrylate (PMMA) and allyl sulfide (AS) fiber composites was fabricated via the electrospinning technique. Fourier Transform Infrared Spectroscopy (FTIR) for functional group identification, X-ray Diffraction (XRD) for crystallinity assessment, Scanning Electron Microscopy (SEM) for surface morphology analysis, Energy-dispersive X-ray spectroscopy (EDX) for elemental distribution analysis, and Brunauer–Emmett–Teller (BET) analysis were used to characterize the fabricated fiber composite materials. The successful interaction between the β-TCP/PMMA/AS fibre composite and crystalline integrity was confirmed by FTIR and XRD investigation. The β-TCP/PMMA/AS exhibited a surface area of 45.410 m^2^/g, a pore volume of 0.059 cm^3^/g, and a pore diameter of 3.9 nm according to BET analysis. *In vitro* cell proliferation assays demonstrated improved osteoblast viability and adhesion compared to control formulations, with nearly 90.0% viability observed at 100 µg. Alizarin Red S (ARS) staining indicated enhanced calcium deposition in the osteoblast formation, confirming the mineralization potential of the β-TCP/PMMA/AS hybrid matrix. These studies show that the β-TCP/PMMA/AS fibre composite has promising potential for bone cell formation, repair/regeneration, and suitability for osteoporosis-related bone tissue engineering applications.

## 1 Introduction

Osteoporosis is a systemic bone disease that is marked by abnormal bone remodeling, resulting in reduced bone mass and microarchitectural deterioration (Lei et al., 2023). Bone remodeling is an intrinsic balance of bone-forming osteoblast cells and bone-resorbing osteoclast cells (Poorirani et al., 2023). If the performance of bone-resorbing osteoclast cells is more predominant than that of bone-forming osteoblast cells, it causes osteoporosis (Poorirani et al., 2023). Osteoporosis has become a significant global medical and societal concern for the aging population, and also more specifically in the case of post-menopausal women (Lei et al., 2023; Poorirani et al., 2023; Hoong et al., 2025). Osteoporosis is called a silent epidemic because of its high prevalence and frequent underdiagnosis. It is predicted to affect nearly 200–500 million people worldwide. About 18.3% of the general population is affected, with a significantly higher occurrence in women (23.1%) compared to men (11.7%). Among those over 50, the prevalence is approximately 6.3% in men and 21.2% in women. The risk of osteoporosis increases steadily with age, reaching nearly 10% in women at age 60, 20% at age 70, 40% at age 80, and up to 66% by age 90. Osteoporosis causes more than 8.9 million fractures each year, leading to abnormal bone remodeling, reduced quality of life, and higher mortality (Keen et al., 2026).

Recently, various methods have been developed to enhance the stability of internal fixation, and one such approach involves systemic administration of anti-osteoporosis drugs (Mukhopadhaya and Bhadani, 2024; Cornell, 2003). Anabolic and anti-resorptive are two conventional drugs were used for the treatment of osteoporosis; one promotes bone formation, and the other reduces bone resorption (Huang et al., 2025). Despite its therapeutic efficacy, adverse effects and long-term adherence issues constrain its effectiveness. Consequently, paving the way for the development of biomaterials with high osteogenic and osteoconductive ability to treat osteoporosis (Lei et al., 2023). The incorporation of bioactive compounds into biomaterials plays a significant role in the regeneration of bone infected with bacteria. Garlic *(Allium sativum Linn)* has various medicinal benefits, including hypolipidemic, hypocholesterolaemia, and antioxidant effects, and can promote bone health (El-Saadony et al., 2024; Sheikh et al., 2025). Organosulfur compounds such as Allyl sulfide (AS), diallyl disulfide (DDS), and diallyl trisulfide (DTS) are found in garlic extract, which possesses anti-oxidants, anti-inflammatory, and cytoprotective properties (Behera et al., 2021). The incorporation of allyl sulfide into the biomaterials will provide an organosulfur compound with known antioxidative, anti-inflammatory, and cytoprotective properties, aimed at enhancing osteogenic potential and cellular response (Ansari and Darvishi, 2024).

Different types of biomaterials have been reported for their ability to improve bone fixation stability. In the realm of orthopedic treatment, bone types of cement have been widely used as fillers to fill irregular trauma sites (Liu et al., 2023). Among bone cements, PMMA poly (methyl methacrylate) is a lightweight, optically clear thermoplastic with high impact material strength (Rahman et al., 2024). It is derived from the methyl methacrylate monomer, a synthetic acrylic polymer. Its properties make it an interesting material for biomedical applications, which include good mechanical properties, weatherability, affordability, ease of processing, less toxicity, minimal inflammatory reactions, and improved fracture resistance (Ivanova et al., 2026; Ramanathan et al., 2024; Gao et al., 2025). However, due to its inertness, it has a slow degradation rate, which hinders bone regeneration. Hence, the development of biodegradable bone cement is crucial for its biomedical applications (Gao et al., 2019).

Biodegradable materials, considered the second generation of biomaterials, play a crucial role in tissue repair and regeneration. These materials undergo gradual degradation within the organism through processes such as dissolution, enzymatic digestion, and cellular phagocytosis, at rates that align with bone formation (Liu et al., 2023). Tricalcium phosphate (TCP) exists in two forms, α-TCP and β-TCP, both of which are bioactive ceramics that serve as bone substitutes due to their exceptional biocompatibility. While β-TCP is created at lower temperatures below 1125 °C, α-TCP is obtained at high temperatures above 1125 °C, and β-TCP is thermodynamically stable in a biological environment (Horch et al., 2006). Among calcium phosphates, β-TCP has been extensively studied for bone regeneration, due to its resorption behaviour, cellular adhesion, and mechanical strength (Ruiz-Aguilar et al., 2018; Tao et al., 2019). As it exhibits not only osteoconductive but also osteoinductive properties, it is highly effective in synthetic bone transplantation (Ruiz-Aguilar et al., 2018). Nowadays, to obtain different morphologies, different preparation methods have been used, resulting in chemical compositions similar to those of natural bone (Ruiz-Aguilar et al., 2018).

In the present study, we have fabricated an AS-incorporated fiber composite for the new bone cell formation for the regeneration of osteoporosis-affected people. Previous studies by Behera et al. (2021) suggest that allyl sulfide, a natural bioactive compound, may be a promising therapeutic target for the treatment of aging-related osteoporosis (Behera et al., 2021). Although the combination of PMMA and β-tricalcium phosphate has been extensively investigated for bone regeneration research, the addition of organosulfur compounds to fibrous ceramic–polymer scaffolds is a relatively new area of investigation. Furthermore, the recognized osteoporosis-associated allyl sulfide, in combination with a bioceramic polymeric fibrous matrix, and the assessment of osteoblast proliferation have not been reported. Since allyl sulfide was incorporated into an electrospun β-tricalcium phosphate (β-TCP)/polymethyl methacrylate (PMMA) fibrous composite for the first time to develop a multifunctional scaffold that combines the osteoconductive properties of β-TCP, the structural support of PMMA, and the potential bioactivity of allyl sulfide. The main goal of this research was to prepare and characterize the β-TCP/PMMA/AS fibrous composite and evaluate its physicochemical properties, morphology, and *in vitro* biocompatibility against osteoblast-like cells. We hypothesize that the allyl sulfide-incorporated β-TCP/PMMA electrospun fiber matrix may enhance osteoblast proliferation, mineralization, and the formation of a bone-mimicking microenvironment, thereby improving its potential for osteoporosis-related bone regeneration in clinical applications.

## 2 Experimental section

### 2.1 Materials

The chemicals were obtained from a commercial manufacturing company. The chemicals obtained are calcium nitrate tetrahydrate (Ca(NO_3_)_2_·4H_2_O), diammonium hydrogen phosphate ((NH_4_)_2_HPO_4_), and Allyl sulfide (C_4_H_8_S) from Sigma-Aldrich, Yan’an, China. Commercially pure Ti foil (Thickness of 0.25 mm) was purchased from Sigma-Aldrich, Yan’an, China. Polymethyl methacrylate (PMMA) was purchased from HiMedia Laboratories, Yan’an, China. All the chemicals, solvents, and other reagents were used as received. Double-distilled (DD) water was used throughout the experiment for washing and experimental reactions.

### 2.2 Synthesis of β-tricalcium phosphate by the wet precipitation method

The previously reported hydroxyapatite preparation method was followed, with slight modifications (Prabakaran et al., 2021), to prepare β-tricalcium phosphate. For the β-TCP preparation, initially 100 mL of 0.02 M diammonium hydrogen phosphate solution and 100 mL of 0.03 M calcium nitrate tetrahydrate solution were prepared separately. Calcium nitrate tetrahydrate solution was slowly added to the phosphate solution using a burette, maintaining the pH at 9.0. The reaction mixture was continuously stirred for 12 h or until a white precipitate formed. The resulting precipitated solution was then filtered using Whatman 41 filter paper and rinsed with double-distilled water, followed by absolute ethanol. The precipitated particles were dried for 48 h at 40 °C, then ground and heated to 900 °C for 2 h. Further, it was collected and cooled at room temperature (25 °C) for the next process.

### 2.3 β-TCP/PMMA and β-TCP/PMMA/AS solution preparation

The β-TCP/PMMA solution was prepared as follows: initially, 400 mg of PMMA was accurately weighed and dissolved in 10 mL of acetone in a sealed 100 mL glass beaker. The beaker was placed on a magnetic stirrer and stirred at room temperature until PMMA was completely dissolved, ensuring a homogeneous solution. Next, 600 mg of β-TCP powder was dispersed in 10 mL of DD water and carefully introduced into the PMMA-acetone solution. The β-TCP and PMMA mixture at a 40:60 wt% ratio was stirred continuously for 15 min to achieve high viscosity, yielding a thick β-TCP/PMMA solution. For the synthesis of the β-TCP/PMMA/AS solution, a separate batch of the β-TCP/PMMA solution was prepared; additionally, 100 mg of Allyl Sulfide (AS) in 2 mL of ethanol was gradually added to the β-TCP/PMMA solution under continuous stirring during preparation. The addition of AS triggered noticeable changes in the solution, resulting in the formation of a β-TCP/PMMA/AS solution. The concentration of AS was fixed based on the previous report demonstrating cytoprotective effects without cytotoxicity and osteogenic properties (Behera et al., 2021).

### 2.4 Electrospinning of β-TCP/PMMA and β-TCP/PMMA/AS fiber

**FIGURE 1:**
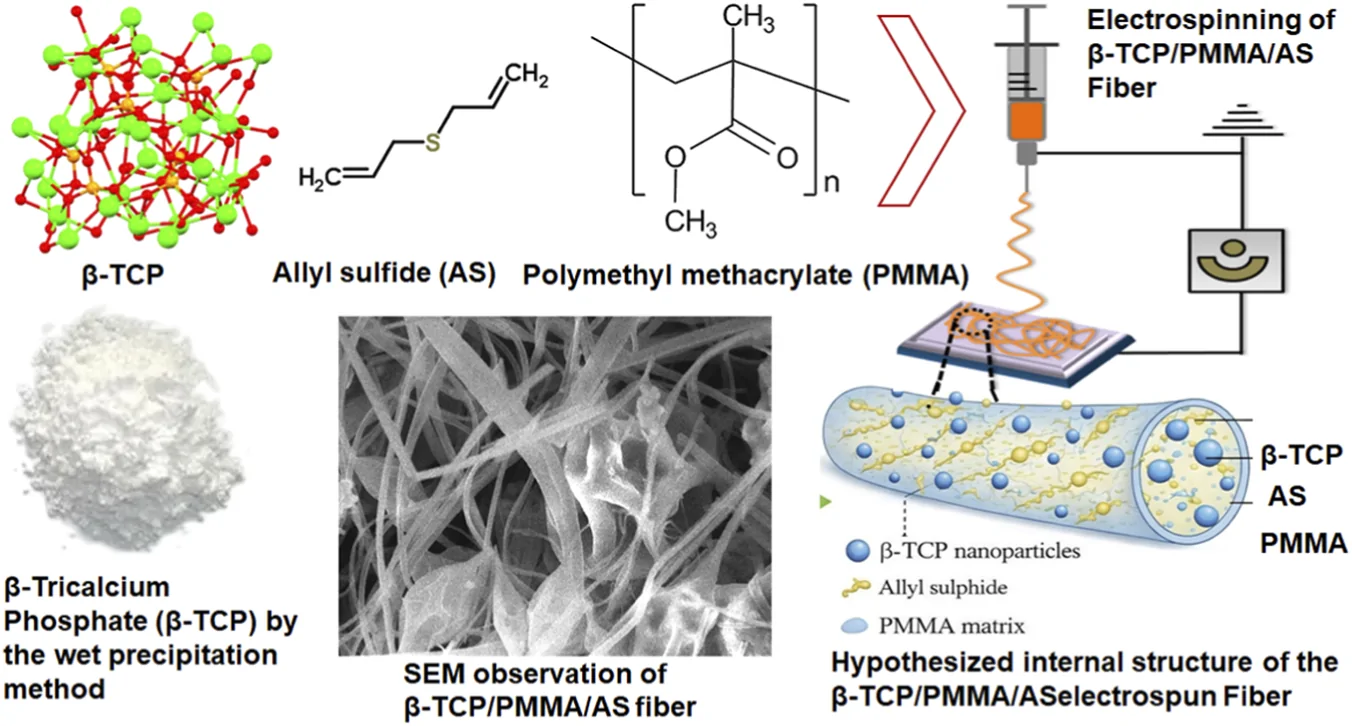
Schematic overview depicting the formation of β-TCP/PMMA/AS fiber through the electrospinning technique.

The β-TCP/PMMA and β-TCP/PMMA/AS solutions were used to fabricate fibers on titanium substrates and on alumina foil via electrospinning technique. Electrospinning was performed using a Royal Electrospinner (Model HD30, 230 V AC, 50 Hz, single phase). The surface of the rotating drum collector, intended for fiber deposition, was covered with aluminium foil to collect the fiber. For coating the fiber on the titanium plate, the surface-treated Ti plate was placed on the alumina foil. Ti-plate coated fiber was used for bone implant preparation of the major bone defect in future investigations. The β-TCP/PMMA spinning solution was loaded into a 5 mL syringe equipped with a metallic needle connected to the spinneret, which was positioned 15 cm from the collector. The electrospinning process was conducted in a closed chamber under a constant voltage of 19.7 kV and a flow rate of 2 mL/h. The procedure was followed by the fabrication of β-TCP/PMMA/AS and β-TCP/PMMA/AS fibers. The schematic of the process for the formation of β-TCP/PMMA/AS fiber via electrospinning is shown in Figure 1.

### 2.5 Physicochemical characterizations

#### 2.5.1 Functional group analysis

The functional group analysis of the prepared materials, such as β-TCP, β-TCP/PMMA, and β-TCP/PMMA/AS fiber, was evaluated from Fourier transform infrared spectroscopy (FT-IR). It was carried out on an IRTRACER-100 (Shimadzu) using the KBr pellet method in the 4000–400 cm^-1^ region.

#### 2.5.2 Phase analysis

The phase composition and crystallinity composition of the prepared material, namely, β-TCP, β-TCP/PMMA, and β-TCP/PMMA/AS fiber, were evaluated by X-ray diffraction (XRD). It was carried out in a Bruker D8 Advance Diffractometer using a monochromatic Cu K source, with a scanning rate (2θ) of 0.02° at a voltage (30 kV) and current (30 mA), and a scan angle from 10° to 60°.

#### 2.5.3 Morphology analysis and BET analysis

The morphology of the prepared material and the minerals present in the fiber composites were assessed using scanning electron microscopy (SEM). The SEM analysis was conducted using a TESCAN VEGA3 SBH instrument under high vacuum and ambient temperature, employing a beam voltage of 5 kV. The dispersed material was coated onto a glass plate, which was then mounted onto an aluminium stub using double-sided carbon tape. Finally, the samples were sputtered with a thin layer of gold before examination. Additionally, the elements present in the samples were determined through EDX analysis. Nitrogen adsorption–desorption measurements were recorded using a Brunauer–Emmett–Teller (BET) surface area analyzer (Anton Paar, Nova 600, Germany), and the specific pore size distribution was evaluated using the Barrett–Joyner–Halenda (BJH) method. The sample was degassed at 100 °C for 10 h.

### 2.6 *In-vitro* biological studies

#### 2.6.1 *In vitro* cell incubation

The MG-63 human osteoblast-like cell line was taken to assess the fabricated fiber materials for their osteogenic potential and it was procured from ATCC, stock cells were cultured in Dulbecco’s Modified Eagle’s Medium (DMEM) supplemented with 10% inactivated Fetal Bovine Serum (FBS), penicillin (100 IU/mL), streptomycin (100 μg mL^-1^) at 37 °C under a humidified atmosphere containing 5% CO_2_ and 95% of air until they reach confluent. The same complete culture medium containing 10% FBS was used during all cellular assays. The cell was dissociated with cell dissociating solution containing trypsin, EDTA, and glucose at 0.2%, 0.02%, and 0.05%, respectively, in PBS. Subsequently, 50,000 MG-63 cells per well were seeded in a 96-well plate and incubated for 24 h at 37 °C in a 5% CO_2_ incubator. After this, the cells were segregated into three groups for analysis of β-TCP, β-TCP/PMMA, and β-TCP/PMMA/AS composite.

#### 2.6.2 Analysis of cell viability and AOES staining study

The cytocompatibility of the prepared materials was evaluated using MG-63 cells, as they are a major *in vitro* test model for their reproducibility and sensitivity to surface-mediated osteogenic effects. The computability of MG-63 cells was assessed through the Methyl Thiazolyl Tetrazolium (MTT) assay. After trypsinization, 100 µL of the diluted MG-63 cell suspension (50,000 cells/well) was added to the 96-well microtiter plate. After 24 h, the supernatant was flicked off, and the plates were washed with medium. β-TCP, β-TCP/PMMA, and β-TCP/PMMA/AS at concentrations ranging from 20 μg mL^-1^–100 μg mL^-1^ were added to the partial monolayer in microtiter plates. Before conducting the biological studies, the samples were sterilized at 121 °C for 15 min under standard pressure conditions. Without the fiber, MG-63 was treated as a control. The plates were then incubated at 37 °C for 24 h in a humidified atmosphere containing 5% CO_2_ and 95% of air for 24 h. After incubation, the test solution was replaced with 100 µL of MTT (5 mg/10 mL of MTT in PBS). And again, the plates were incubated for 4 h at 37 °C in a humidified atmosphere containing 5% CO_2_ and 95% air. The supernatant was removed, and 100 µL of DMSO was added, and the plates were gently shaken to solubilize the formed formazan. The absorbance was measured using a microplate reader at a wavelength of 590 nm. The percentage growth inhibition was calculated using the specified formula, and the concentration of the test drug required to inhibit cell growth by 50% (IC_50_) was determined from the dose-response curves for each cell line (Denizot and Lang, 1986). An indirect extract-based cytotoxicity assay was performed according to ISO 10993–5. Cell viability and membrane integrity were evaluated using acridine orange/ethidium bromide (AO/EB) dual fluorescence staining. MG-63 cells cultured on 100 μg mL^-1^ β-TCP, β-TCP/PMMA, and β-TCP/PMMA/AS composites were washed with PBS and stained with AO and EB solutions each for 5 min at room temperature. AO permeates intact cell membranes and stains viable cells green, whereas EB enters cells with compromised membranes, emitting orange-red fluorescence. Fluorescence images were captured using a fluorescence microscope, and at least five random fields per sample were analyzed to qualitatively assess cell viability and morphology (Kasibhatla et al., 2006; Liu et al., 2015).

$$\text{Cell viability }\left(\%\right)=100\;–\;\left(\text{OD of sample}/\text{OD of Control}\right)\;x\;100.$$

#### 2.6.3 Analysis of calcium deposition using the Alizarin Red S (ARS) strain

Calcium deposition mineralization was assessed using ARS staining in this study for all three samples, namely, β-TCP, β-TCP/PMMA, and β-TCP/PMMA/AS, at a concentration of 100 μg mL^-1^ in 1-day, 7-day, and 14-day cultures of MG-63 cells. At each time point, 2 mL of PBS with a pH of 7.2 was used for washing the wells, and they were fixed with formalin (4%) at room temperature for around 15 min. Following this, 1 mL of ARS stain, which has a pH between 4.1 and 4.3, was incubated at room temperature with orbital shaking. The spent ARS was depleted from the wells, and the wells were washed with distilled water without removing any calcium phosphate matrix. The stained monolayers were visualized and photographed using phase-contrast inverted microscopy, with mineralization appearing as a deep red stain. Based on the red strain color, the calcium mineral deposition and osteogenic potential were qualitatively observed (Prabakaran et al., 2021).

### 2.7 Statistical analysis

All quantitative experimental data are reported as the study’s mean ± standard deviation (SD), obtained from three independent experiments performed in triplicate. Mean data were determined from the results using one-way analysis of variance (ANOVA), followed by Tukey’s *post hoc* multiple comparison test to assess statistical significance among the various groups. The results were interpreted by plotting a graph, and the statistical significance was assessed at p < 0.05.

## 3 Results and discussion

### 3.1 FTIR analysis

**FIGURE 2:**
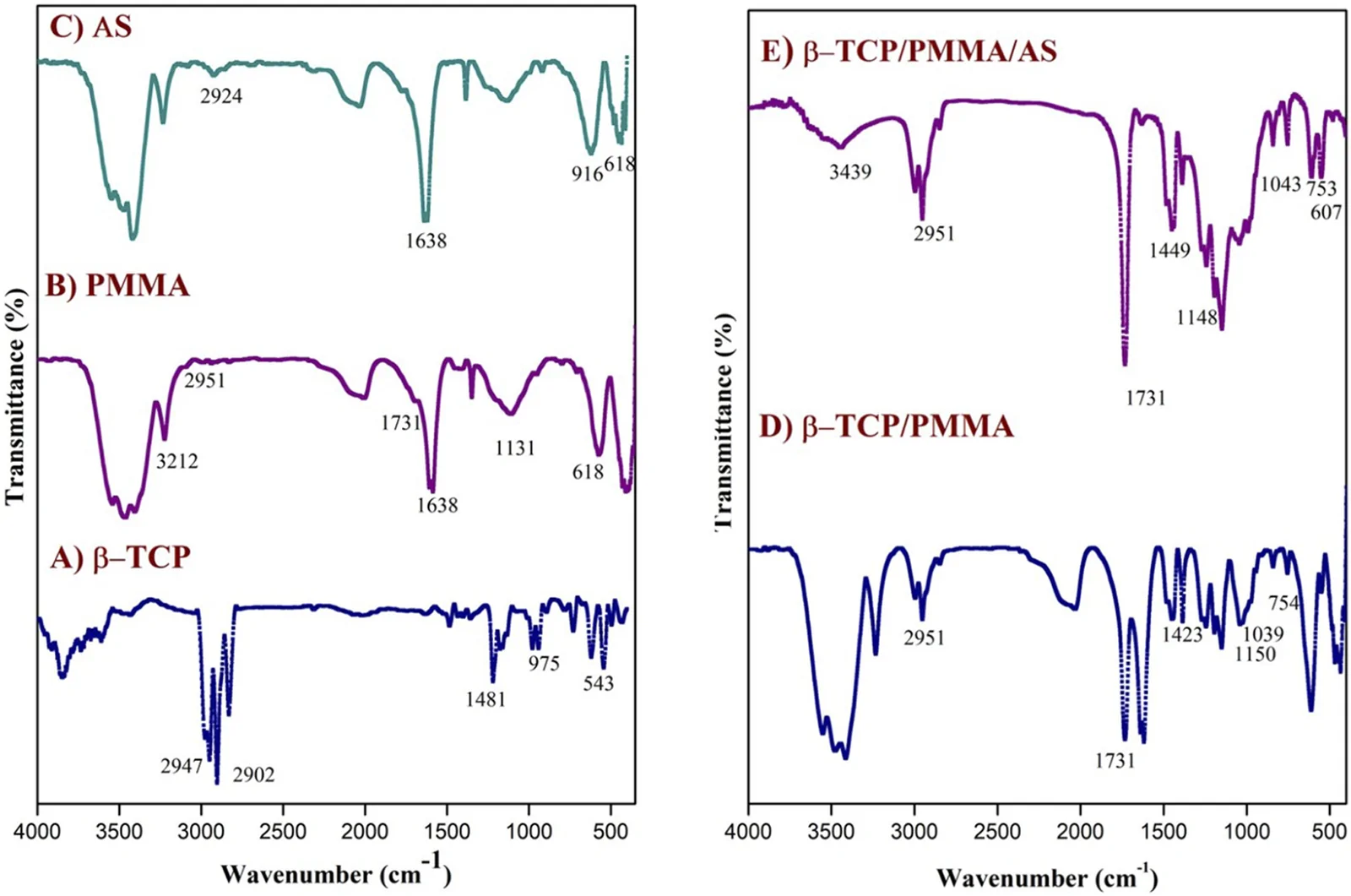
FT-IR results spectrum of **(A)** β-TCP, **(B)** PMMA, **(C)** AS, **(D)** β-TCP/PMMA, and **(E)** β-TCP/PMMA/AS fiber composites.

The FTIR spectra of β-TCP, PMMA, AS, and their composites were investigated, and their results are presented in Figure 2. The β-TCP spectrum shows characteristic phosphate (PO_4_^3-^) peaks at 975 and 543 cm^-1^ was observed and it shows the consistent with reported β-TCP structures. A weak band near 1481 cm^-1^ is attributed to carbonate substitution within the β-TCP lattice (Figure 2A) (Abdelrazek et al., 2020). In the FTIR spectrum of PMMA exhibits characteristic vibrational features, including the band at ∼3212 cm^-1^ is linked to OH stretching from adsorbed moisture, while the peak at 2951 cm^-1^ is associated with CH stretching vibrations of methyl groups and a strong ester carbonyl (C=O) stretching band at ∼1730 cm^-1^, and COC stretching vibrations in the 10001200 cm^-1^ region were noted. (Figure 2B). The AS FTIR spectrum shows characteristic absorption bands at 2924 cm^-1^ due to the aliphatic C–H stretching, 1638 cm^-1^ attributed to the C=C stretching of the allyl group, and additional bands at 916 cm^-1^ and 618 cm^-1^ were observed in Figure 2C. The β-TCP/PMMA composite spectrum shows that the characteristic phosphate bands of β-TCP are retained but with reduced intensity, which can be attributed to the dominant absorption of the polymer phase and the relative proportion of components. Such intensity variations are commonly observed in the polymer and ceramic composites due to differences in phase distribution and signal contribution (Roohani-Esfahani et al., 2010). The ester carbonyl (C=O) band of PMMA around 1731 cm^-1^ remains observable in the composite, although its relative intensity and resolution differ compared to pure PMMA, likely due to variations in local environment and phase dispersion within the composite matrix. Additional bands at 2951 cm^-1^ are due to C–H stretching, 1423 cm^-1^ is assigned to CH3 bending, and the range 1150 to 1039 cm^-1^ corresponds to C–O–C stretching, further confirming the presence of the polymer phase. Low-wavenumber bands at 754 cm^-1^ and 607 cm^-1^ correspond to phosphate bending modes of β-TCP component, indicating that the ceramic phase remains structurally stable after composite formation (Kankala et al., 2018). In the β-TCP/PMMA/AS composite, the spectra show combined features of all components, including phosphate bands of β-TCP and characteristic polymer vibrations (Verma et al., 2006). The preservation of β-TCP peaks indicates that the crystal structure remains intact after composite formation. Overall, the FTIR results confirm the coexistence of β-TCP, PMMA, and AS phases, with no clear evidence of new chemical bond formation, suggesting that composite formation is primarily governed by physical mixing and phase distribution.

### 3.2 SEM, EDAX, and mapping analysis

**FIGURE 3:**
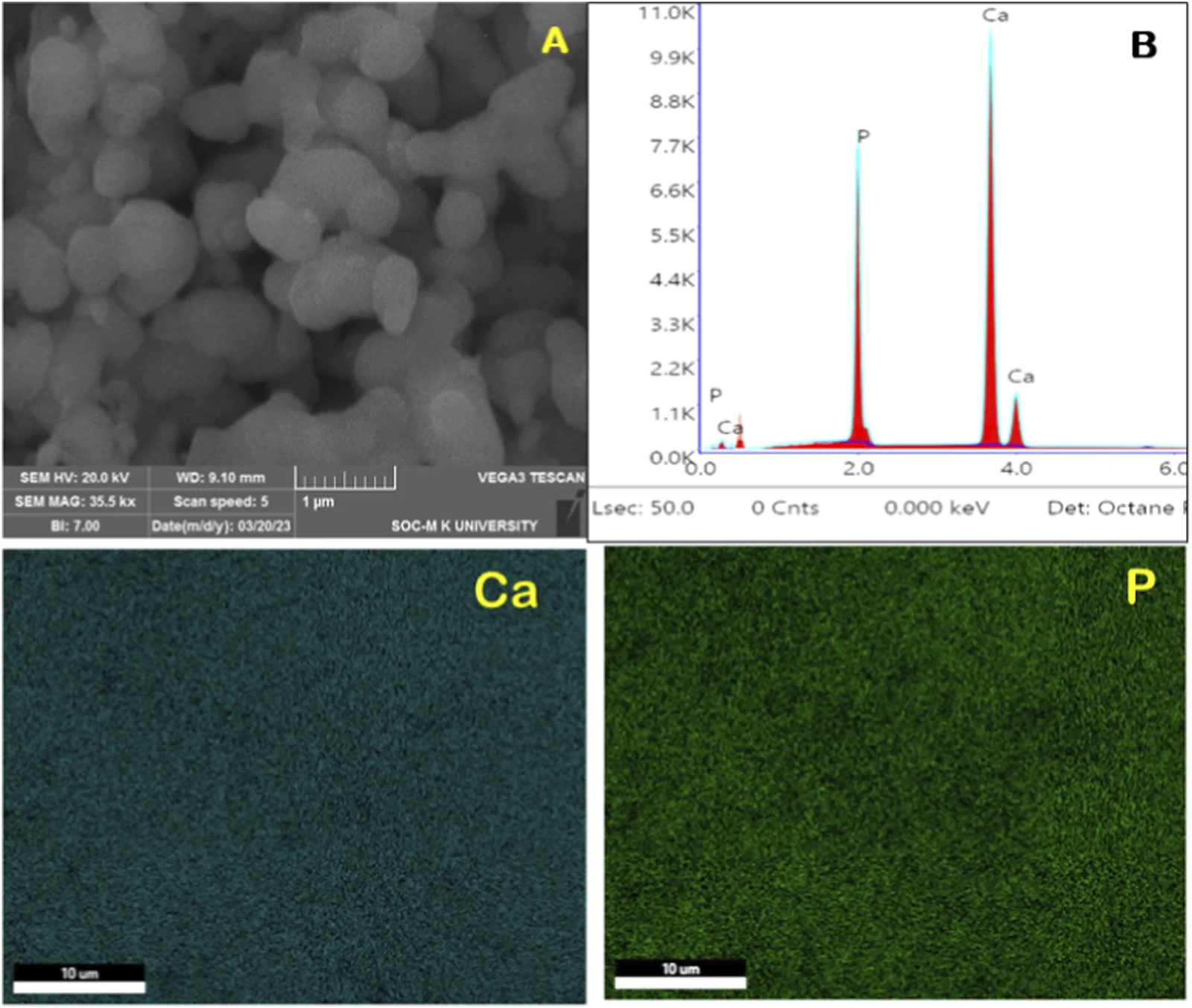
SEM morphology β-tricalcium phosphote **(A)** with their EDX **(B)** & calcium and phosphote distribution in the β-tricalcium phosphote ceramic.

SEM analysis was performed to evaluate the morphology of all prepared samples. Figure 3A shows the β-TCP morphology observation, as it exhibits a uniform agglomerated morphology with a uniform size and spherical particles. The presence of major elements such as calcium and phosphorus associated with β-TCP is confirmed by EDAX analysis. The semi-quantitative analysis yielded a Ca/P stoichiometric atomic ratio of β-tricalcium phosphate of Ca/P ≈ 1.50, which closely matches the theoretical value for stoichiometric β-TCP (Ca_3_(PO_4_)_2_). The elemental distribution on the β-TCP particles was investigated using elemental mapping analysis, with a focus on calcium and phosphorus. A uniform distribution of both ions was observed in the samples, as shown in Figure 3. Elemental mapping images further demonstrated a homogeneous spatial distribution of Ca and P throughout the sample, confirming compositional uniformity and phase purity of the β-TCP. Thus, it further confirms the presence and spatial arrangement of these elements, providing valuable insights into the structural formation of the β-TCP particles.

**FIGURE 4:**
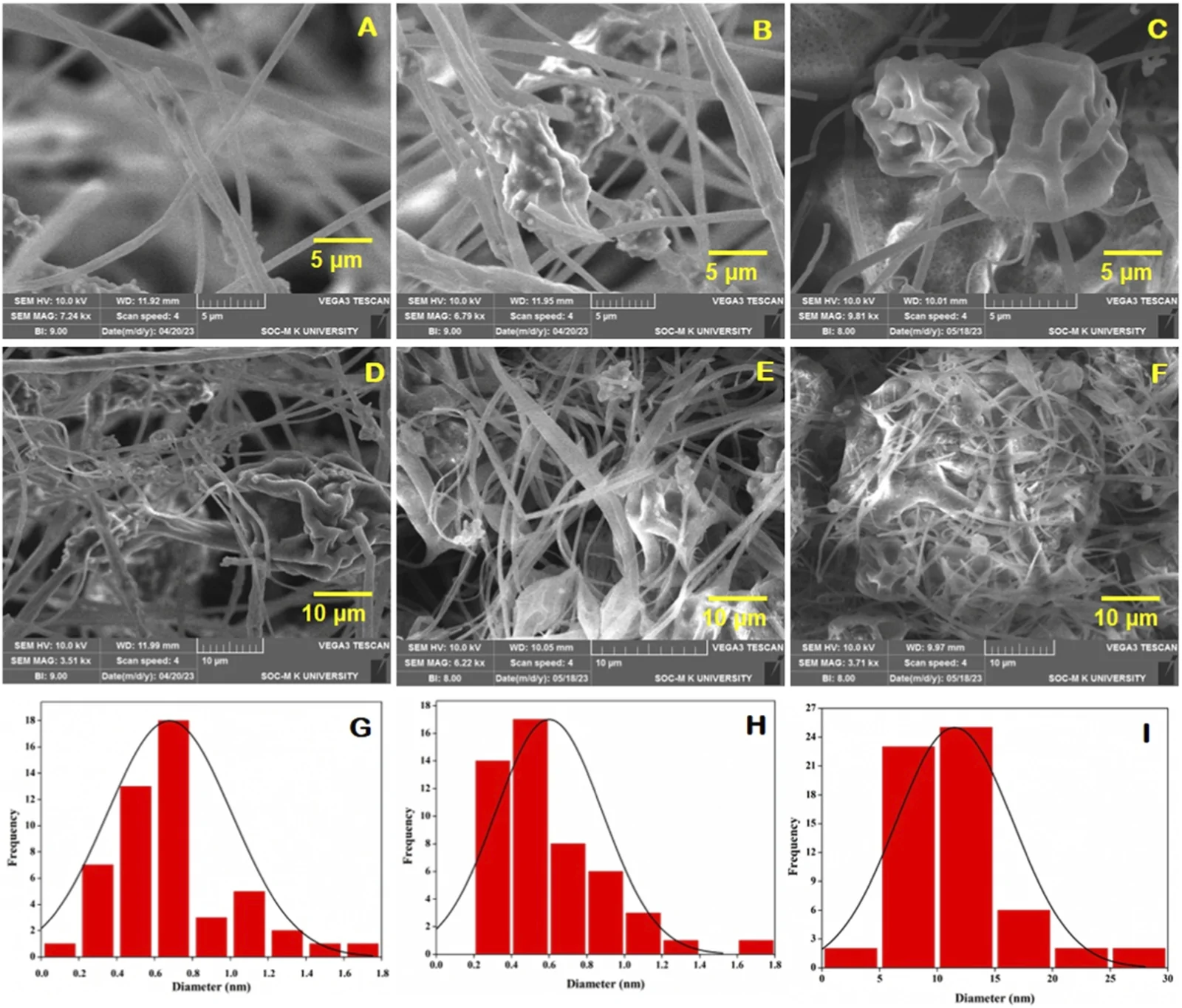
SEM morphology of β-TCP/PMMA **(A)**, β-TCP/PMMA/AS in the alumina foil **(B)**, and β-TCP/PMMA/AS fiber composite in the titanium plate **(C)**, and their quantitative fiber diameter of β-TCP/PMMA **(D,G)**, β-TCP/PMMA/AS in the alumina foil **(E,H)**, and β-TCP/PMMA/AS fiber composite in the titanium plate **(F,I)**.

The β-TCP/PMMA and β-TCP/PMMA/AS composite were coated on alumina foil and a titanium plate to fabricate an electrospun fiber composite to promote cell adhesion, migration, and proliferation (Yao et al., 2019). Figure 4 shows the β-TCP/PMMA and β-TCP/PMMA/AS fibers deposited on alumina foil and a titanium plate Figure 4A indicates the β-TCP/PMMA fiber, Figure 4B, shows β-TCP/ PMMA/AS coated alumina foil, and the β-TCP/PMMA/AS fiber composite in the titanium plate morphology is presented in Figure 4C. The diameter of the fiber of β-TCP/PMMA, β-TCP/PMMA/AS on alumina foil, and β-TCP/PMMA/AS fiber composite on a titanium plate were quantitatively determined from SEM image (Figures 4D–F). All three electrospun fibers show a straight, uniform, interconnected network, after the AS incorporation in the β-TCP/PMMA identified particles in the fiber. These interconnected porous mimics of the extracellular matrix ultimately enhanced osteoblast proliferation (Song et al., 2021). Compare β-TCP/PMMA/AS fibers in alumina foil and titanium deposited fibers that are angulated to form a fiber ball. The average fiber diameter of β-TCP/PMMA and β-TCP/PMMA/AS fiber of alumina was determined from these images using ImageJ software. The β-TCP/PMMA composite exhibited a mean fibre diameter of 0.7 μm, indicating relatively uniform fibre formation (Figure 4G). The same β-TCP/PMMA composite coated on aluminium foil showed a reduced mean diameter of 0.5 μm (Figure 4H), suggesting enhanced fibre refinement, possibly due to favourable substrate fibre interactions. Upon incorporation of allyl sulfide (AS) and coating on a titanium plate, the mean diameter increased to 11.4 μm (Figure 4I), indicating pronounced fibre thickening, likely arising from changes in solution properties and intermolecular interactions during composite formation. From the slight variation, it can be confirmed that the presence of allyl sulphide influences the dynamics of fiber formation. The porosity shown in the morphology confirms that the fibers have a well-connected structure, which is essential for nutrient diffusion and cell infiltration. Preliminary mechanical characterization based on the previous reports suggests that the β-TCP/PMMA/AS fibers may possess adequate tensile strength and stiffness for bone scaffold applications; a comprehensive quantitative evaluation is currently underway and will be presented in future work. These results are in line with earlier studies on β-TCP/PMMA fibrous composites by Gao et al. (2019), which showed tensile strengths of 4–6 MPa and Young’s moduli of 70–90 MPa (Gao et al., 2019). By strengthening the mechanical integrity bond between the β-TCP particles and the PMMA polymer matrix, the addition of allyl sulfide is anticipated to improve mechanical integrity. All findings indicate that the electrospun β-TCP/PMMA/AS composite possesses the strength, flexibility, and porosity required for successful bone tissue engineering applications.

### 3.3 XRD analysis

**FIGURE 5:**
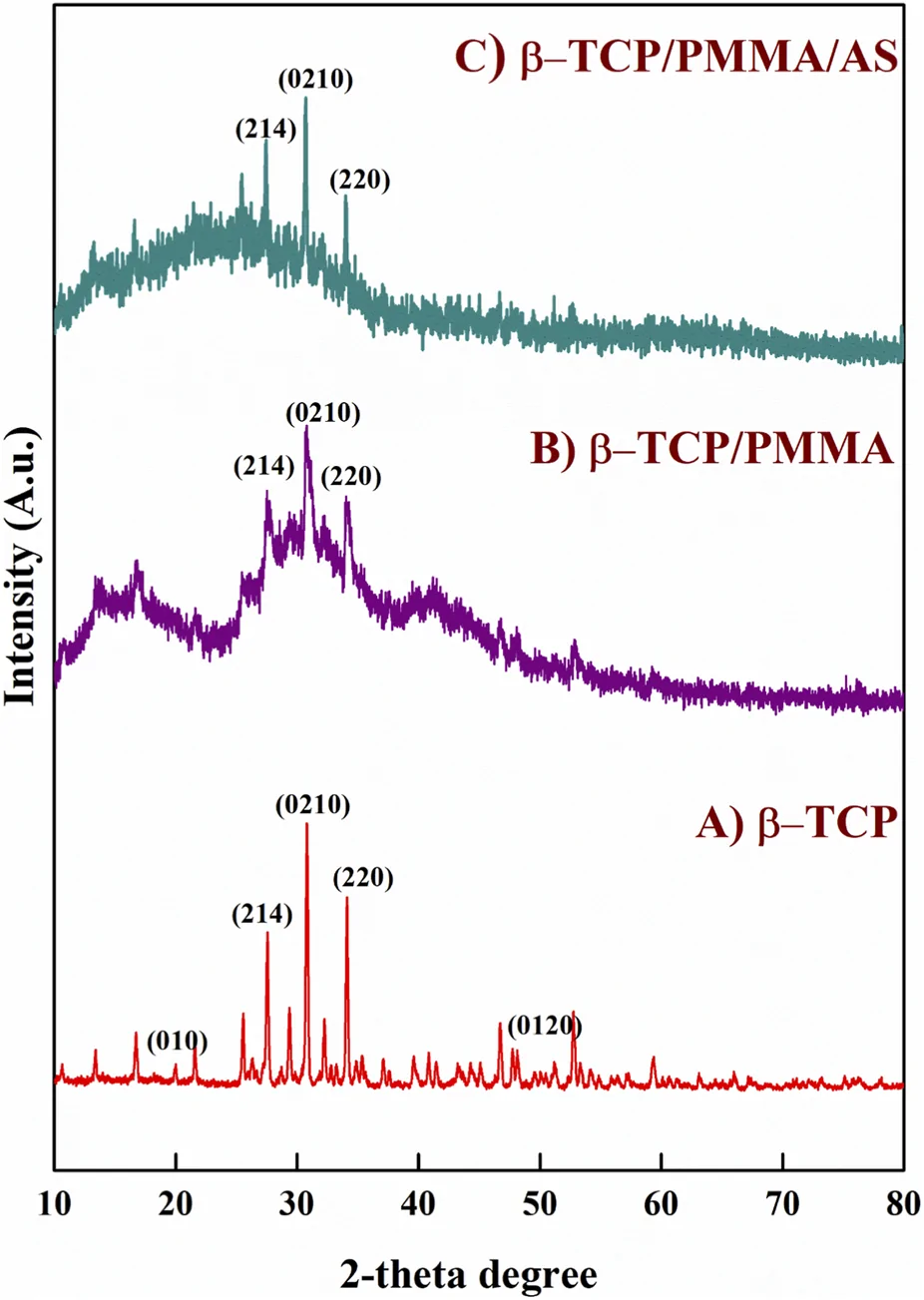
The XRD diffraction analysis of β-TCP **(A)**, β-TCP/PMMA **(B)**, and β-TCP/PMMA/AS **(C)** fiber composites.

X-ray diffraction analysis of the β-TCP, β-TCP/PMMA, and β-TCP/PMMA/AS fibrous composite collected from the alumina foil was performed using a diffractometer, with a copper anticathode providing radiation. The XRD diffraction pattern of β-TCP, β-TCP/PMMA, and β-TCP/PMMA/AS fiber composites was presented in Figure 5. By investigating the X-ray intensities of calcium and phosphorus at the implant-bone interface, it is possible to detect the presence of the Ca-P-rich layer. The XRD pattern shows characteristic diffraction peaks of β-tricalcium phosphate at approximately 2θ ≈ 27.8°, 31.0°, 34.4°, and 50.3°, corresponding to the (214), (0210), (220), and (0120) crystallographic planes, respectively, which are consistent with the standard β-TCP phase (JCPDS No. 09–0169) (Avinashi et al., 2025; Wojcik et al., 2024). The planes were decreased after the PMMA incorporation into the β-TCP composite, as depicted in Figure 5B. The addition of amorphous PMMA polymer to a β-TCP ceramic reduces diffraction pattern intensities by acting as a diluent and decreases the overall fraction of the crystalline structure. Additionally, the PMMA polymer linkage disrupts the long-range order of the β-TCP crystalline lattice, resulting in decreased peak intensities. Furthermore, the 2θ angle values were approximately 27.4°, 30.6°, and 33.9°, with the plane value of (0210) increased in the β-TCP/PMMA/AS composite. It is due to the incorporation of AS molecules in the composite. The addition of AS to the β-TCP/PMMA composite makes interfacial interactions, such as weak coordination with Ca^2+^ ions and secondary interactions with the polymer phase. These can influence the internal structural arrangement, leading to minor variations in peak intensities and positions without altering the fundamental β-TCP crystal structure. These results have helped elucidate the nature and materials chemistry of β-TCP ceramics, PMMA polymer, and AS, as well as their crystalline properties, which could contribute to the development of biomaterials and their biomedical applications.

### 3.4 Surface area, pore size, and pore volume analysis

**FIGURE 6:**
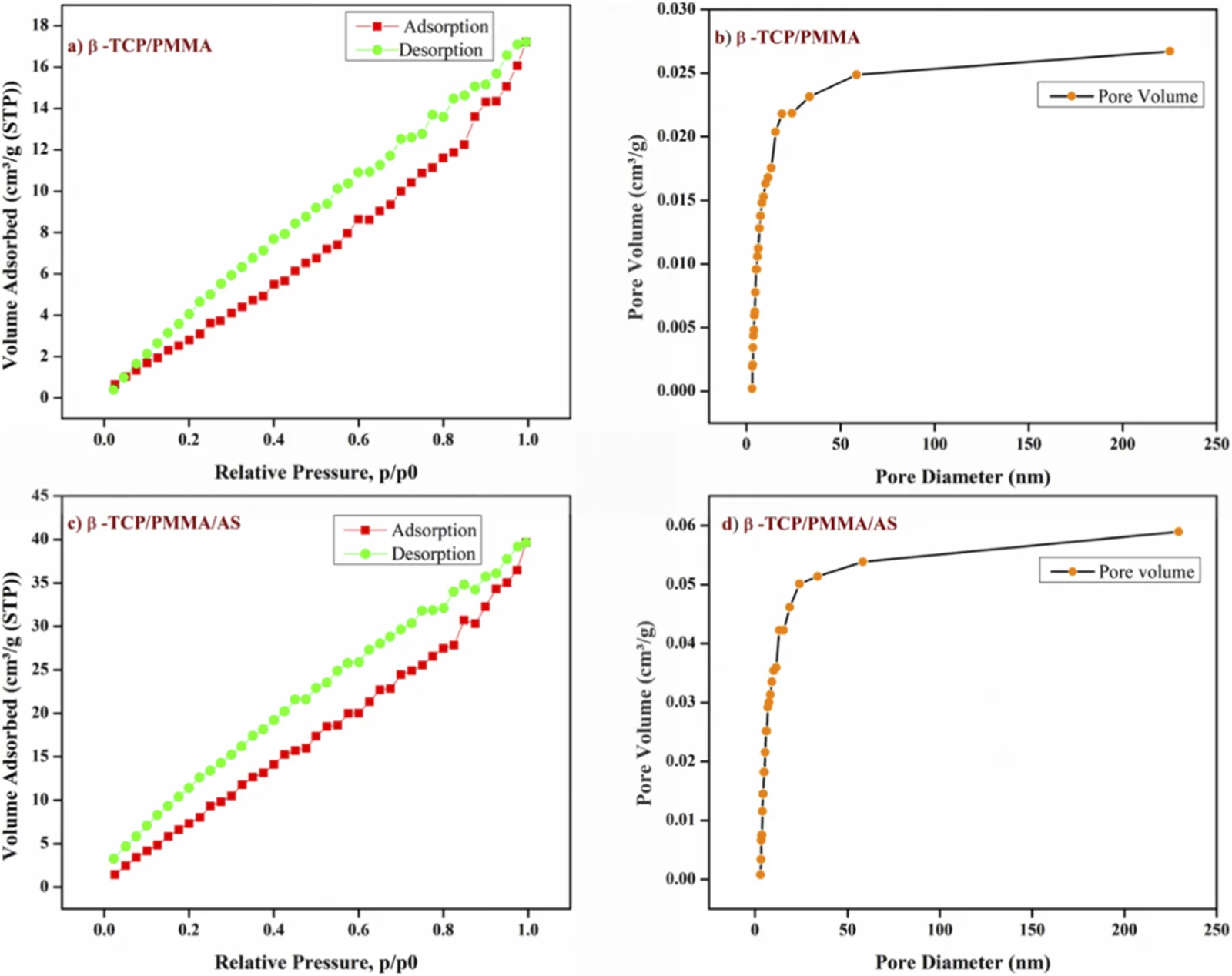
The Surface area, pore size, and pore volume analysis of β-TCP/PMMA **(a,d)** and β-TCP/PMMA/AS **(c,d)** fiber composites.

The nitrogen adsorption–desorption isothermal curves and pore size distribution of β-TCP/PMMA and β-TCP/PMMA/AS were investigated, and the results were presented in Figure 6. Figure 6a shows the surface area of 16.777 m^2^/g of β-TCP/PMMA, with a type IV isotherm, whereas Figure 6b represents the BJH pore size distribution of β-TCP/PMMA, indicating a mesoporous structure in the range of 2–50 nm and a pore diameter of 3.143 nm. It is crucial for tissue regeneration applications. Similarly, the nitrogen adsorption–desorption isothermal curves and pore size distribution of β-TCP/PMMA/AS are shown in Figures 6c,d, indicating an increased surface area of 45.410 m^2^/g, a pore volume of 0.059 cm^3^/g, and a pore diameter of 3.9 nm. The addition of AS significantly alters the texture and acts as a structural modifier, resulting in nearly a threefold increase in surface area. These results are well correlated with the SEM morphology observation. The presence of a Type IV isotherm and mesoporous structure indicates a biomimetic structure that can promote rapid nucleation in physiological fluids. Furthermore, the increased surface area enhances ion exchange, which plays a critical role in bone regeneration by facilitating the signaling processes required for osteogenesis (Wa et al., 2024). The synergistic combination of enhanced surface area and well-defined microporosity, therefore, positions the β-TCP/PMMA/AS composite as a promising candidate for bone tissue engineering applications.

### 3.5 Cytocompatibility analysis

**FIGURE 7:**
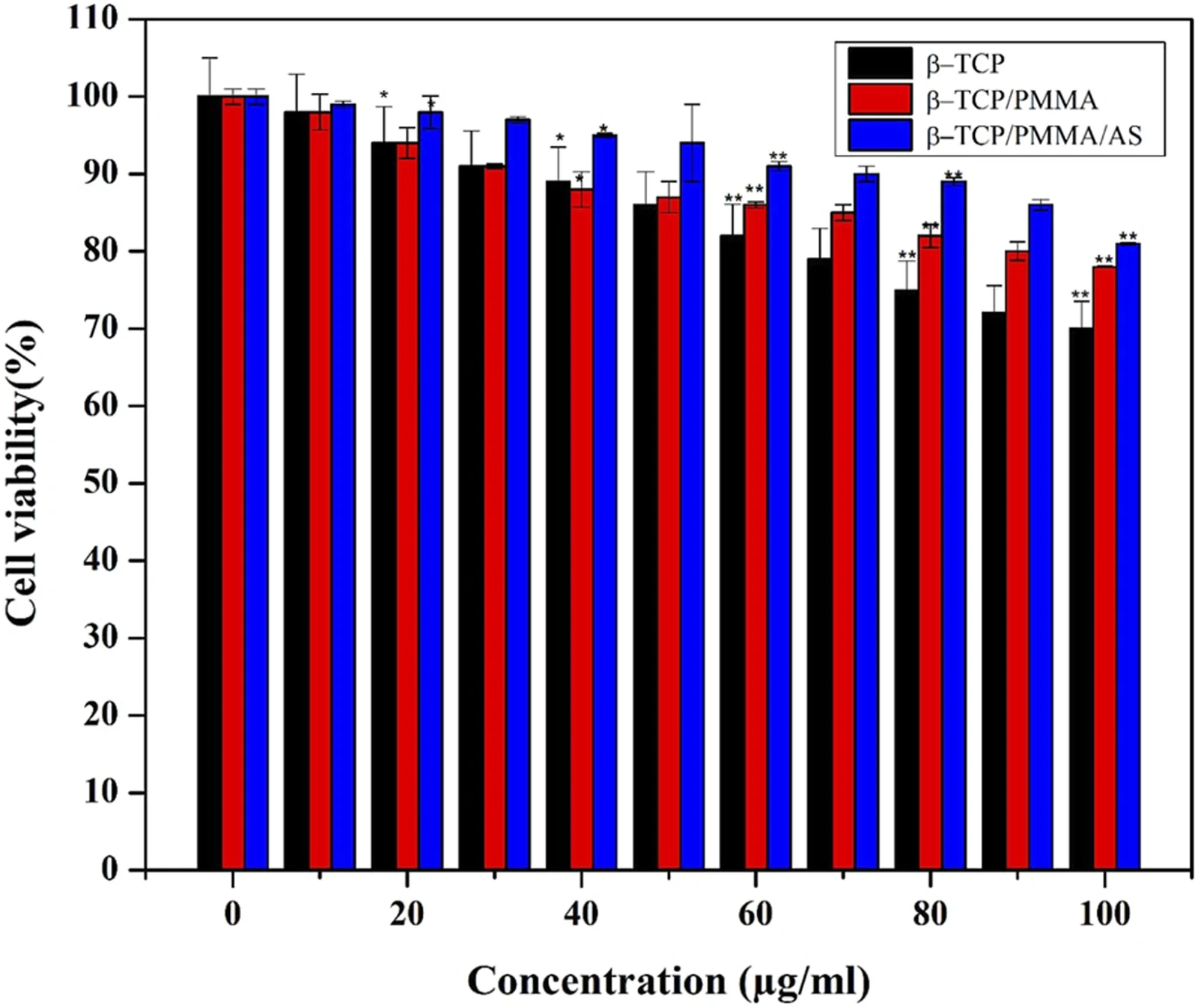
*In vitro* cytocompatibility assessment of β-TCP, β-TCP/PMMA, and β-TCP/PMMA/AS composites evaluated by MTT assay after 24 h incubation at different concentrations.

Cytocompatibility of β-TCP, β-TCP/PMMA, and β-TCP/PMMA/AS composites was evaluated using the MTT assay method with 24 h of incubation on MG-63 cells at different concentrations 10 μg to 100 μg, and the result of the cell viability is given in Figure 7. At lower concentrations, all three samples exhibited high cell viability, indicating they cause no toxic effects with good cytocompatibility. With increasing concentration, a gradual reduction in cell viability was observed for all samples; however, the viability values remained above the cytotoxic threshold, confirming the non-toxic nature of the materials. Among the tested samples, β-TCP/PMMA/AS consistently exhibited higher cell viability than β-TCP and β-TCP/PMMA at all concentrations. At a 100 μg concentration of β-TCP/PMMA/AS, nearly 90.0% of cell viability was observed, and with the same concentration, β-TCP/PMMA and β-TCP samples show 81.0% and 70.0%, respectively. Since all three composites exhibited >70% cell viability, the β-TCP/PMMA/AS composite demonstrated significantly higher cell viability than β-TCP and β-TCP/PMMA at higher concentrations (p < 0.05). These results suggest that the enhanced cellular response can be attributed to the presence of allyl sulfide, which is known for its antioxidant and cytoprotective properties, promoting favorable cell–material interactions. The incorporation of PMMA further enhances surface compatibility, thereby supporting osteoblast adhesion and proliferation. Similar enhancements in MG-63 cell proliferation have been reported for bioactive composite systems containing polymers and biofunctional additives, such as chitosan, bioactive glass, and organosulfur compounds (Hu et al., 2012; Prabakaran and Rajan, 2021; Wu et al., 2019). The PMMA/AS and pure AS were not evaluated independently for osteogenic contribution, because PMMA alone is biologically inert and lacks osteoconductive properties, and AS has been reported to modulate oxidative stress and inflammatory signaling pathways. The present study demonstrated a synergistic enhancement of the osteoblast response within a β-TCP-containing composite, rather than the isolated effect of AS. Although many researchers are studying bone regeneration, MG-63 osteoblast-like cells, derived from osteosarcoma, may not fully mimic the behavior of mesenchymal stem cells or primary osteoblasts in osteoporotic bone environments. The proliferation rate is higher in MG63 cells, and it altered the signaling metabolism. Overall, the MTT assay results confirm that the β-TCP/PMMA/AS composite contains components that are cytocompatible and suitable for bone tissue engineering and regenerative applications in osteoporosis.

### 3.6 Live/death assay analysis

**FIGURE 8:**
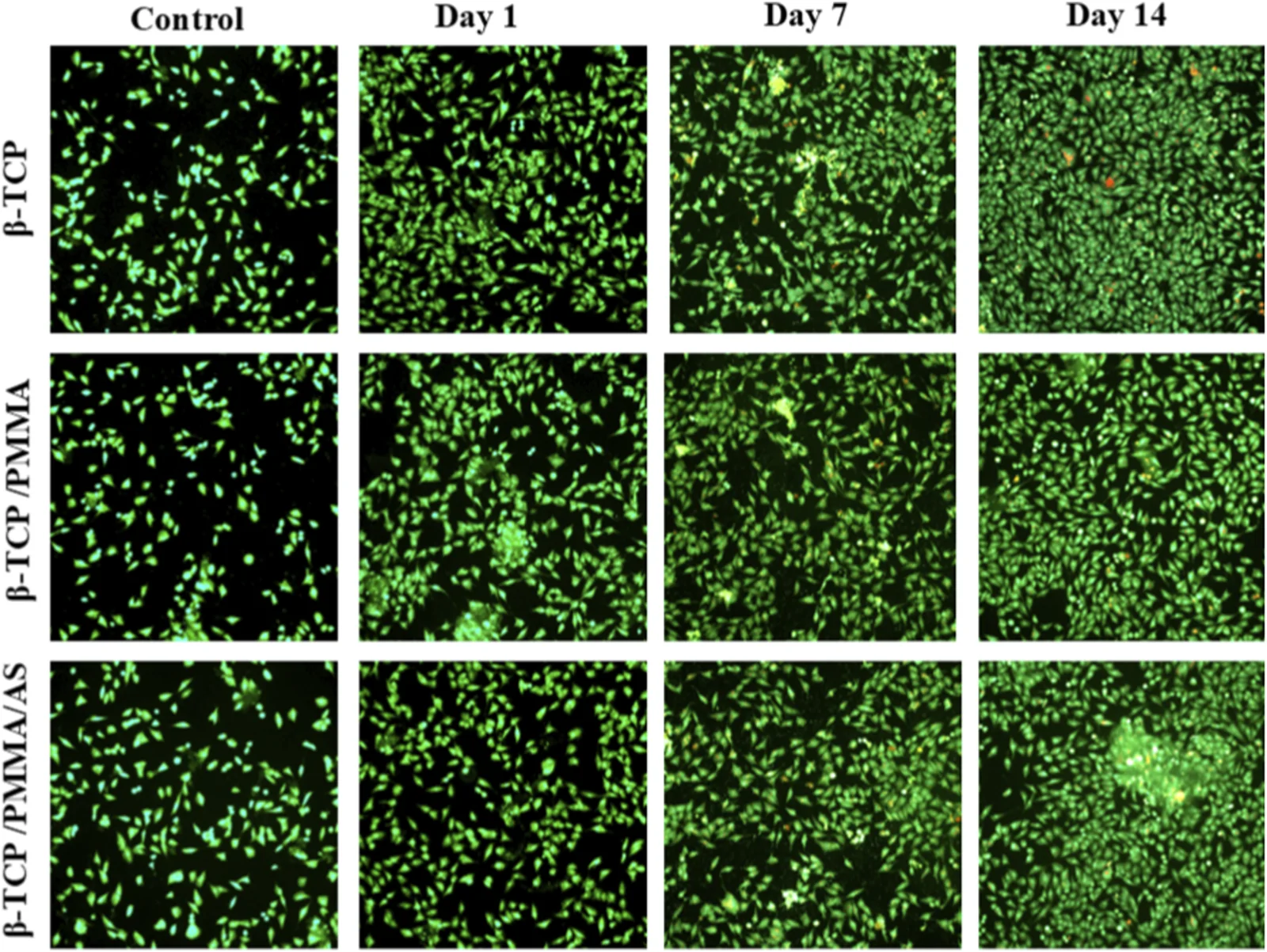
Fluorescent images of AO/EB-stained MG-63 cells treated with β-TCP, β-TCP/PMMA, and β-TCP/PMMA/AS after 1, 7, and 14 days of incubation.

The dual stain, Acridine Orange/Ethidium Bromide (AO/EB), is used to obtain fluorescence micrographs, which are then used to evaluate the survival and apoptotic morphology of MG-63 osteoblast-like cells cultured on β-TCP, β-TCP/PMMA, and β-TCP/PMMA/AS composites. AO strain passes through both live and dead cells and binds to DNA, giving green fluorescence, whereas EB strain passes through only dead cells, giving orange-red fluorescence. Because of these properties, live cells appear green, early apoptotic cells appear yellowish-green, and dead cells appear orange-red under the microscope. From Figure 8, it is clear that all three samples indicate good cell viability and low cytotoxicity, as they emit a green colour. However, the β-TCP/PMMA/AS shows a light red-orange background and denser cell attachment due to the combined PMMA and AS components. Occasionally, a few orange-red fluorescent cells were observed, possibly indicating a small group of late-apoptotic or dead cells, or background staining. The dominant green fluorescence across all samples indicates that the scaffolds support cell survival, aligning with the high viability observed in the MTT assay. It aligns with the earlier work by Behera et al. (2021), which showed that allyl sulfide and similar sulfur compounds help protect cells by reducing harmful reactive oxygen species (ROS) and supporting bone cell formation and mitochondrial health (Behera et al., 2021). The combination of β-TCP and PMMA helped form a structure resembling the natural extracellular matrix (ECM) of bone, providing favorable sites for cell attachment and mechanical flexibility to support cell growth. According to recent research by Lei et al. (2023) and Lu et al. (2021), antioxidant-enriched biomaterials dramatically increase osteoblast viability in oxidative environments by modifying redox-sensitive signalling pathways. Overall, the observed fluorescence pattern confirms that β-TCP/PMMA/AS fibers support cellular viability and promote a positive redox milieu, thereby enhancing osteogenic potential. Together, these results show that the synergistic interaction of AS, PMMA, and β-TCP lowers oxidative stress and promotes osteoblastic activity, both of which are necessary for efficient bone tissue regeneration (Lei et al., 2023; Lu et al., 2021). In the current study, the cytocompatibility and live/dead cell analyses were performed over a short incubation period at selected concentrations. While the results showed good cytocompatibility and more live cells, the investigation also included a higher screening concentration, a longer incubation period, and quantitative live/dead cell counts to better assess efficacy and osteogenic performance.

### 3.7 Calcium deposition using Alizarin Red S (ARS) straining

**FIGURE 9:**
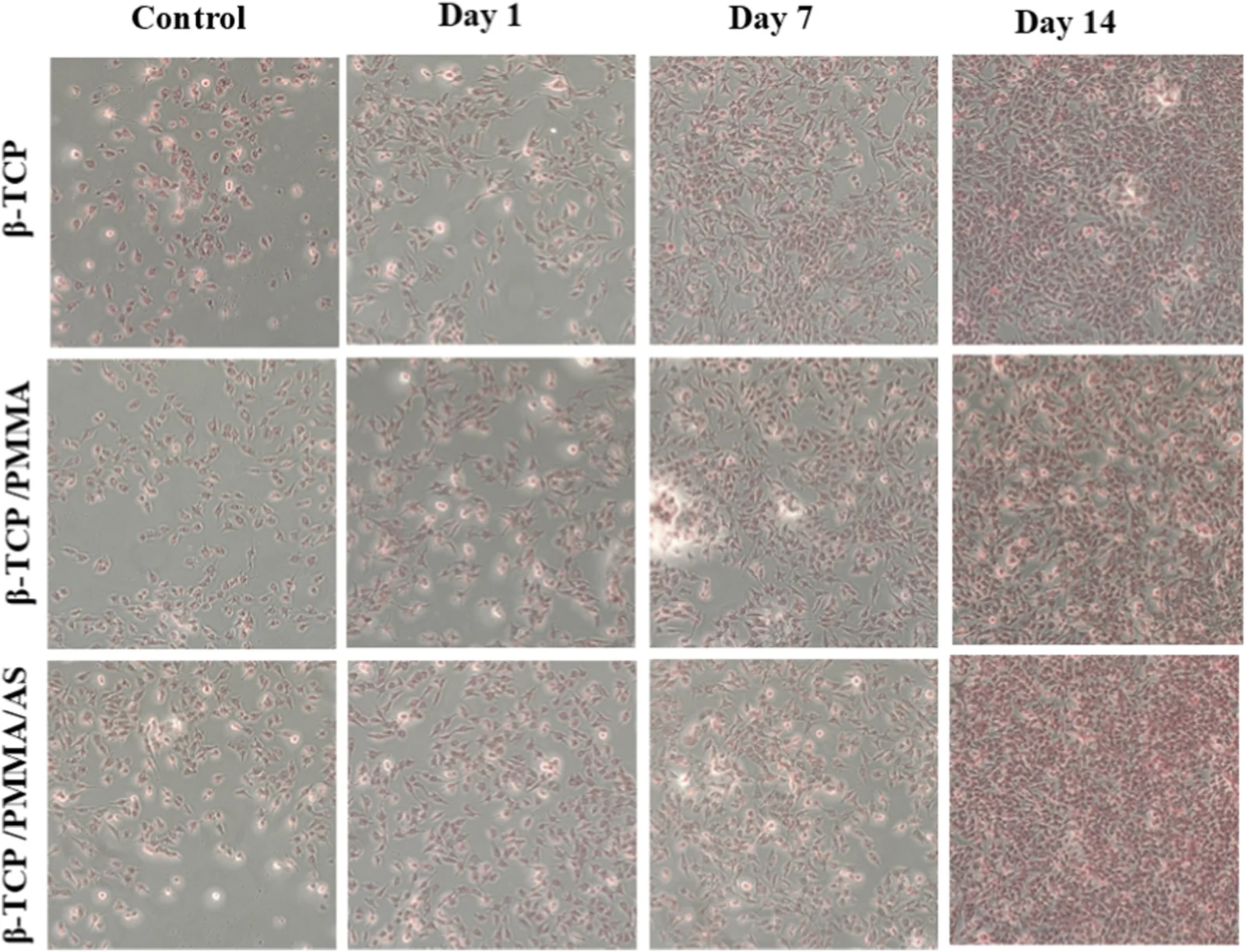
Optical microscopic morphology of MG-63 osteoblast-like cells stained with ARS solution at control, 1, 7, and 14 days of treatment with β-TCP, β-TCP/PMMA, and β-TCP/PMMA/AS fibrous composites.

The MG-63 cells demonstrated successful adhesion and viability on the β-TCP, β-TCP/PMMA, and β–TCP/PMMA/AS fiber composites from 1 day to 14 days, as evidenced by cellular staining with ARS. Osteogenic differentiation, as assessed by ARS staining, induced calcium precipitation within the fiber composites, and the elevation of ECM calcium content was shown to significantly promote osteogenic differentiation (Sumathra and Rajan, 2019; Elakkiya et al., 2023). Figure 9 depicts the cells treated with β-TCP, β-TCP/PMMA, and β-TCP/PMMA/AS at 100 μg mL^-1^ with the optical microscopic morphology of MG-63 osteoblast-like cells. The trend toward Ca^2+^ mineralization was observed at 1, 7, and 14 days, and the aggregates with different red stains were observed after composite treatments, whereas minimal staining was observed on the first day. This demonstrates the efficacy of promoting both calcium deposition and osteogenic differentiation, particularly with β-TCP/PMMA/AS fiber composite. The staining intensity increased with time, reaching the highest level on day 14. The red staining of β-TCP/PMMA/AS fiber was always thicker than the others, implying a higher calcium ion concentration on the cell’s surface. Qualitative ARS staining was used to confirm calcium deposition, despite the moderate red colour observed in the microscopic field in Figure 9. The significant mineralisation with β-TCP/PMMA/AS, as it exhibits a denser red colour in calcium content, compared to the β-TCP/PMMA and β-TCP after 14 days. The relatively weak red colour may be attributed to a uniformly distributed mineralisation process rather than distinct macroscopic aggregates. Significantly, these results show that incorporating PMMA and AS does not impair mineralisation and may enhance osteogenic differentiation. The synergistic action of minerals such as β-TCP, PMMA, and AS could promote osteogenic differentiation (Sangeetha et al., 2018). These results demonstrate that the β-TCP/PMMA/AS scaffold effectively promotes calcium deposition and osteogenic differentiation, even in the absence of prominent nodular formations, consistent with early-stage mineral nucleation. Meanwhile, the quantitative ARS staining combined with statistical analysis would provide more exact results of calcium deposition along with longer drugation studies, such as 21 and 28 days. Therefore, in the future investigation will focus on achieving quantitative calcium mineralization and extended culture periods to further validate the osteogenic potential of the fabricated scaffolds.

## 4 Conclusion

In the present study, we investigated and characterized electrospun fibrous β-TCP, β-TCP/PMMA, and β-TCP/PMMA/AS for applications in osteoporosis-affected bone. FT-IR analysis confirmed the presence of characteristics of functional groups associated with the calcium, phosphate, PMMA, and AS in the fiberous composite. XRD analysis confirmed the crystalline nature of β-TCP, with a transition to amorphous phases upon the addition of PMMA and AS in β-TCP/PMMA and β-TCP/PMMA/AS, respectively. Morphological observations demonstrated well-distributed fibrous structures that provide a favorable microenvironment for cellular attachment and proliferation. Surface area and porosity results confirm the feasibility of promising materials for bone regeneration applications. *In vitro* cell viability studies with MG-63 cells showed good viability across all three composites, with β-TCP/PMMA/AS exhibiting the highest viability among the other composites. Importantly, the fiberous composite retained cell viability above 80% even at the highest tested concentration (100 μg mL^-1^), indicating the prepared materials' good cytocompatibility according to ISO-10993–5 guidelines. The calcium deposition and osteogenic differentiation studies using ARS staining demonstrated enhanced mineralization in β-TCP/PMMA/AS compared with the other formulations. The investigation determined that incorporating PMMA and allyl sulfide improves the biological performance of β-TCP-based composite without compromising mineralization. Finally, the β-TCP/PMMA/AS composite shows promise as a bioactive fibrous scaffold for bone tissue engineering applications. Future studies will focus on detailed quantitative mineralization analysis, long-term cell culture evaluations, and *in vivo* validation to further assess its clinical translatability.

## Data Availability

The original contributions presented in the study are included in the article/supplementary material, further inquiries can be directed to the corresponding author.
